# *Pneumocystis jirovecii* Pneumonia in a Patient with Rheumatoid Arthritis Treated with Abatacept

**DOI:** 10.1155/2014/835050

**Published:** 2014-09-17

**Authors:** Fabio E. Ospina, Andrés Agualimpia, Fabio Bonilla-Abadía, Carlos A. Cañas, Gabriel J. Tobón

**Affiliations:** ^1^Instituto de Investigaciones Clínicas, Fundación Valle del Lili, Cali, Colombia; ^2^Fundación Valle del Lili, Cra 98, No. 18-49, Cali, Colombia

## Abstract

Rheumatoid arthritis (RA) is an autoimmune disease characterized by synovial membrane inflammation and joint cartilage destruction. Abatacept is a biologic agent that blocks the costimulation signals, preventing antigen presentation and proliferation of T lymphocytes. It is approved for the treatment of patients with RA. *Pneumocystis jirovecii* pneumonia (PJP) is an infectious disease complicating several immunosuppressive drugs. PJP associated with abatacept has not been reported yet in the medical literature. Various factors, such as the mechanism of action of abatacept, may contribute to predisposing to  *Pneumocystis jirovecii* infection. In this paper, we report a patient with RA who developed PJP under abatacept treatment.

## 1. **Introduction**

Rheumatoid arthritis (RA) is an autoimmune disease characterized by synovial membrane inflammation and joint cartilage destruction [1–3]. The pathogenesis of the disease is complex, involving different cell populations of the immune response [4]. Due to a better understanding of the pathophysiology, multiple biological drugs have been approved for use in patients with RA [5].

Among these drugs, abatacept (CTLA-4Ig) has been widely used in RA patients. It is a selective costimulatory modulator, formed by a human soluble recombinant fusion protein (extracellular domain of human CTLA-4 and a hinge fragment of the Fc portion of human IgG1), which block costimulation induced by binding of the CD80/CD86 from the antigen presenting cells and the CD28 from T lymphocytes. Abatacept inhibits T lymphocytes activation and proliferation [6, 7]. This drug has been approved for the management of RA by FDA [4, 8].

The most common side effects related to the abatacept treatment are headache in 18% of patients, pharyngitis (12%), dizziness (9%), cough (8%), back pain (7%), and hypertension (7%) [9, 10]. The incidence of infections per 100 patients per year is 0.60 (95% CI from 0.47 to 0.76), being pneumonia, urinary tract infection, soft-tissue infections, bronchiolitis [9], and nosocomial pneumonia the most common [11].

*Pneumocystis jirovecii *(*P. jirovecii*) is one of the most important opportunistic pathogens in immunosuppressed patients [12]. With the advent of biological therapy, cases of community-acquired pneumonia by* P. jirovecii* have increased in recent years. The most associated biologic DMARDs with* P. jirovecii* pneumonia (PJP) are infliximab [13], although some cases associated with rituximab treatment have also been reported in the medical literature [14]. In this paper, we report a case of PJP associated with the use of abatacept in a patient with RA.

## 2. Case Report 

A 64-year-old female patient with a previous history of 8 years of seronegative-RA was admitted to our emergency department with a clinical picture of 4 days characterized by dry cough, fatigue, weakness, fever (38.5°C), and dyspnea. Before admission to our institution, she was evaluated in a primary care center, where azithromycin and levofloxacin treatment were indicated, with no improvement.

In the previous clinical history, the diagnosis of seronegative-RA was based on negative test for both rheumatoid and anticyclic citrullinated peptide antibodies. In addition, another clinical diagnosis such as systemic lupus or Sjögren's syndrome was excluded (antinuclear antibodies and antiextractable nuclear antigens were negative and sicca symptoms were absent). She was treated initially with sulfasalazine, methotrexate, and prednisolone for almost two years. By refractory disease, a biological therapy with adalimumab 40 mg every 15 days was initiated, and seven months later this treatment was withdrawn by an allergic reaction. Thus, abatacept (750 mg IV every month) in association with methotrexate (15 mg weekly) was initiated four years ago, whereby the patient shows improvement of symptoms.

On admission to the emergency room, physical examination revealed a poor general status, with respiratory distress, tachycardia (110 beats for min), and fever (38.1°C). Her BP was 118/74 mmHg. Cardiopulmonary and abdominal physical examination did not show relevant findings. Articular examination showed no inflammatory signs.

Extensive studies were completed. Laboratory test showed a normal white blood count (7.790/*μ*L), without neutrophilia (5.830/*μ*L) or lymphopenia (1.140/*μ*L). Hemoglobin (12.7 g/dL) and platelets (276.000/*μ*L) count were normal. Chemical liver and kidney function were normal. Urine test was also normal. LDH values were elevated 417.00 U/L (normal range 135–214 U/L). Arterial blood gases showed hypoxemia and acute phase reactants were elevated (C-reactive protein 11.20 mg/dL, normal level to 0.5 mg/dL, and eritrosedimentation rate of 89 mm/hour). Blood cultures and detection of influenza viruses A, B, and A subtype H1N1 2009, Histoplasma antigen in urine, and Cryptococcus, Gram, smear, KOH, acid alcohol resistant microorganisms, Romanowsky Wright, and deep mycosis blood culture were all negative.

Due to severe pulmonary involvement, bronchoscopy and bronchoalveolar lavage were performed, where the methenamine silver staining was positive for* P. jirovecii.*

**Figure 1:**
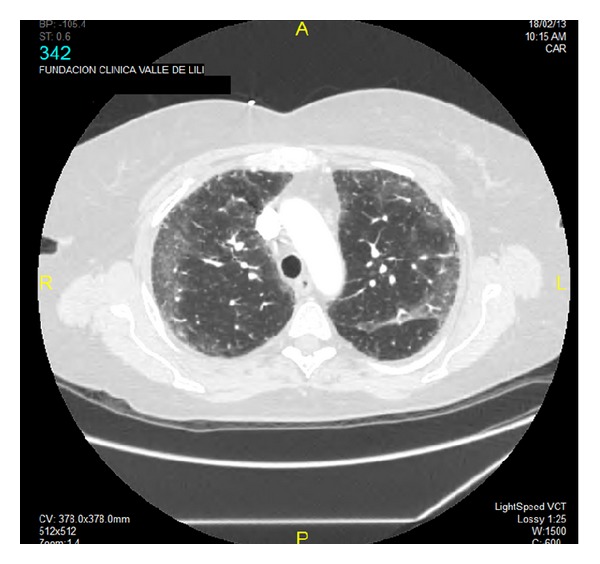
Chest computed tomography showing diffuse reticular interstitial infiltrates with ground-glass pattern of nonspecific interstitial pneumonia in a rheumatoid arthritis patient with* Pneumocystis jirovecii *pneumonia.

Lung imaging showed diffuse reticular interstitial infiltrates in both lungs on X-ray and interstitial infiltrates with ground-glass pattern of nonspecific interstitial pneumonia on CT scan (Figure 1).

The patient was transferred to the intensive care unit, where treatment was given initially with noninvasive ventilatory support and methylprednisolone. Due to the immunosuppression status, an empirical treatment for viral and atypical bacterial pneumonia was initiated with levofloxacin 500 mg QD, oseltamivir 75 mg BID, and amantadine 100 mg BID. After the diagnosis of PJP, the initial management was discontinued and coverage began with trimethoprim/sulfamethoxazole 320/1600 mg TID and clindamycin 900 mg TID (which was suspended for adverse drug reaction). After 11 days of hospitalization and improvement of ventilatory and infectious goals, she was discharged with ambulatory treatment to complete 21 days of trimethoprim/sulfamethoxazole.

The patient recovered well, without evidence of infectious relapse. Abatacept and methotrexate were completely suspended. Regular follow-up in the rheumatology service has been instaured. Treatment is conducted with prednisolone 5 mg QD, without active RA.

## 3. Discussion

A host-adequate immune system status is necessary to establish a good response to infection. Healthy subjects with normal antigen presenting cells (APCs) and lymphocyte function can establish an adequate immune response leading to the destruction of the fungus after inhaling particles of* P. jirovecii* [15].

On the contrary, in patients treated with immunomodulatory agents such as abatacept, the activation of T cells both CD8+ and CD4+ by the APC is compromised, and in turn the activation of CD4+ lymphocytes leads to reduced activation of B cells [4, 6]. Specifically, abatacept modulates the monocytes phenotype, influencing the transendothelial migration and migratory capacity by decreasing the expression of adhesion molecules [16]. All these disturbances lead to a decrease of the immune response to a microorganism injury [4, 16].

PJP has been reported in the medical literature in several cases of patients treated with different biologic agents, including infliximab and rituximab [13, 14], as well as patients treated with other drugs such as methotrexate. Although some cases of PJP related to abatacept have been reported in the FDA registries, their outcome has not been described. Here, we report a case of PJP related to abatacept treatment. Our patient was treated also with methotrexate that may increase the risk to present PJP. Thus, this case should call attention to doctors treating this type of patients. The PCP is a potentially fatal condition in immunocompromised hosts. For this reason, a high suspicion of this infectious complication must be kept in mind in order to establish an early detection and treatment in patients with RA managed with DMARDs. Finally, more studies are necessary in these patients to thereby establish recommendations for the treatment and prophylaxis for PCP.
